# I329L protein-based indirect ELISA for detecting antibodies specific to African swine fever virus

**DOI:** 10.3389/fcimb.2023.1150042

**Published:** 2023-06-07

**Authors:** Zhiyong Shen, Wenchen Qiu, Haorui Luan, Chunxi Sun, Xinya Cao, Gang Wang, Jun Peng

**Affiliations:** ^1^College of Veterinary Medicine, Shandong Agricultural University, Tai’an, China; ^2^Breeding Management Department, Beisanxia Husbandry Company Limited, Hegang, China; ^3^Shandong Provincial Key Laboratory of Animal Biotechnology and Disease Control and Prevention, Shandong Agricultural University, Tai’an, China

**Keywords:** African swine fever virus, I329L protein, prokaryotic expression, indirect ELISA, antibody detection

## Abstract

African swine fever (ASF) is a disease that causes severe economic losses to the global porcine industry. As no vaccine or drug has been discovered for the prevention and control of ASF virus (ASFV), accurate diagnosis and timely eradication of infected animals are the primary measures, which necessitate accurate and effective detection methods. In this study, the truncated ASFV I329L (amino acids 70–237), was induced using IPTG and expressed in *Escherichia coli* cells. The highly antigenic viral protein I329L was used to develop an indirect enzyme-linked immunosorbent assay (iELISA), named I329L-ELISA, which cut-off value was 0.384. I329L-ELISA was used to detect 186 clinical pig serum samples, and the coincidence rate between the indirect ELISA developed here and the commercial kit was 96.77%. No cross-reactivity was observed with CSFV, PRRSV, PCV2, or PRV antibody-positive pig sera, indicating good specificity. Both intra- assay and inter-assay coefficients were below 10%, and the detection sensitivity of the iELISA reached 1:3200. In this study, an iELISA for ASFV antibody detection was developed based on the truncated ASFV I329L protein. Overall, the I329L-ELISA is a user-friendly detection tool that is suitable for ASFV antibody detection and epidemiological surveillance.

## 1 Introduction

African swine fever (ASF) is an acute, febrile and highly contagious infectious disease caused by ASF virus (ASFV), which affects domestic pigs and wild boars of various ages and breeds, leading to significant deaths and economic losses. Notably, highly virulent strains can have a fatality rate of 100%. This disease is notifiable to the World Organization of Animal Health and is listed as a class I animal disease in China (Sánchez et al., 2013; Luo et al., 2017). ASF was first discovered in Kenya in 1921, introduced to Western Europe and Latin America successively in 1957, appeared in the Caucasus region in 2007, and then rapidly spread to Russia, Eastern Europe, and neighboring countries (Gogin et al., 2013; Alonso et al., 2018; Gaudreault et al., 2020). ASFV is a DNA virus with a capsular membrane. It has a genome length ASFV of 170–190 kb and a linear double-stranded DNA. The genome comprises a central conserved region of 125 kb in the middle and complementary variable regions at both ends, and contains approximately 175 open reading frames that encode over 50 structural and 100 nonstructural proteins (de Oliveira et al., 2011; Golnar et al., 2019).

ASF was first reported in Liaoning Province in August 2018, from where it subsequently spread to 31 provinces in China within half a year, causing substantial economic losses and had a devastating impact on pig production in China (Li et al., 2022). During this epidemic, the virulence of some strains decreased, and natural recombinant low-pathogenicity strains emerged in China. Although the clinical symptoms and pathological changes in infected pigs have changed considerably, they dramatically still pose a major threat to the pig industry. In recent years, some infected pigs have survived both subacute and latent infections. Infected pigs generally have high antibody levels; however, the virus is generally undetectable in the blood as well as nasal and oral secretions after viremia. Therefore, ASFV infection cannot be reliably monitored by detecting the virus in blood samples, nasal swabs or mouth swabs. The development of an antibody detection method based on the early expression of viral replication proteins is conducive to the timely and accurately monitor antibody levels in infected pigs.

pI329L is a relatively conserved sequence, a type I transmembrane protein encoded by ORF I329L, which is expressed late in viral infection and is mainly distributed on the cell membrane of infected cells and the surface of the viral vesicle membrane (Greiner, 1995). To date, no ELISA has been reported for this protein. In this study, the I329L protein was used as a detection target. The truncated I329L protein was expressed and purified, and an indirect ELISA method was established to detect ASFV antibodies, which can aid in improved diagnosis and epidemiological investigation of ASFV.

## 2 Materials and methods

### 2.1 Strains, plasmids, sera and animals

DH5α competent cells (Takara, Dalian, China) were used for plasmid propagation and cloning. *Escherichia coli* BL21 (DE3) competent cells (Transgen biotech, Beijing, China) were used as the expression hosts. pET–32a (+) was used as the expression plasmid in *E. coli*. pEGFP–C1 vector was used for protein expression in mammalian cell lines. ASFV-positive and ASFV-negative serum samples were provided by China Animal Health and Epidemiology Center (Qingdao, China). BALB/c mice were purchased from Shandong Province Experimental Animal Center (Jinan, China).

### 2.2 Construction of the recombinant I329L

The I329L gene was relative conserved. I329L sequences of 100 strains from Africa, Europe, Central Asia, Russia, East Asia, Southeast Asia, Australia, and South America during 2018-2022 were collected and compared for their homology. The results showed that 92 strains were 98% to 100% homology, 6 strains were 90% to 98% homology, and 2 strains were less than 90% homology, and the last two strains are from Russia. Among them, 20 strains from China have 100% homology. The gene design software DNASTAR Protean was used to analyze the hydrophilicity, hydrophobicity, secondary structure, and antigenicity of the I329L protein, which encodes the I329L protein of ASFV strain China/2018/AnhuiXCGQ (GenBank no. MK128995.1), based on its amino acids. It was determined that the fragment corresponding to amino acids 70-237 had good hydrophilicity and antigenicity (**Supplementary Figure 1**). The nucleotide sequences of the truncated fragment were synthesized and cloned into the T clonal vector, pBR-ASFV-tI329L (Tsingke, Qingdao, China), in which the restriction sites of the enzymes *BamH* I and *Xho* I were added to both ends of the aforementioned segment. The full-length *I329L* gene with the same restriction sites was also synthesized and constructed as pBR-ASFV-fI329L. The amplicon was cloned into pET-28a with *BamH* I and *Xho* I restriction enzyme sites. Subsequently, the recombinant plasmids were transformed into DE3 for overnight incubation at 37°C in a kanamycin-treated agar plate. The recombinant plasmids were then extracted and confirmed by DNA sequencing (Sangon Biotech, Shanghai, China).

### 2.3 Expression and purification of the recombinant I329L protein

The recombinant I329L protein was expressed in prokaryotic cells induced by 0.5 mM IPTG at 37°C for 6 h. SDS-PAGE analysis was performed to examine the I329L protein after cell lysis, and the obtained protein was purified using a Ni-NTA resin-based column as described previously (Li et al., 2022). Subsequently, the purified I329L protein was identified *via* western blot using ASFV-positive serum as the primary antibody.

### 2.4 Western blot analysis

Purified I329L proteins were separated by SDS–PAGE and transferred to a PVDF membrane (Millipore, Darmstadt, Germany) as described previously (Guinat et al., 2016). After blocking with PBST containing 5% BSA for 2 h, the transferred proteins were incubated with ASFV-positive serum for 2 h. The membrane was then incubated with HRP-conjugated goat anti-pig secondary antibody (Beyotime Biotechnology, Shanghai, China) for 1 h. Protein bands were visualized using Clarity Western ECL substrate (Bio–Rad) with NcmECL Ultra (NCM Biotech, Suzhou, China).

### 2.5 I329L pAbs production

Six-week-old female BALB/c mice were intraperitoneally injected with 100 µL of antigen emulsified with 100 µg purified I329L protein and incomplete Freund’s adjuvant (Sigma-Aldrich, Shanghai, China). Freund’s adjuvant was mixed according to the manufacturer’s instructions with an equal volume of protein to form an emulsion. Three immunizations were performed every two weeks at the same dosage. Ten days after the final immunization, the inoculated mice were euthanized, and sera were collected for subsequent examination.

### 2.6 Indirect immunofluorescent assay

The antigenicity of I329L was determined using indirect immunofluorescence assay (IFA). Briefly, the full-length *I329L* gene (pBR-ASFV-fI329L) was inserted into the pCAGGS-EGFP vector *via* enzyme digestion and a ligand reaction (pCAGGS-EGFP-fI329L). Subsequently 293T cells were transfected with the eukaryotic expression plasmid pCAGGS-EGFP-fI329L at approximately 60% concentration using ExFect Transfection Reagent (Vazyme, Nanjing, China) according to the manufacturer’s instructions. After fixing and blocking with 4% paraformaldehyde fixative and 5% BSA, the cells were subsequently incubated with the aforementioned mouse serum and TRITC-labeled goat anti-mouse IgG (Vazyme, Nanjing, China), at 1:300 dilution at 37 °C for 1 h. The specific fluorescence was visualized using a fluorescence microscope (Leica, Buffalo Grove, IL, USA).

### 2.7 Indirect ELISA

Purified I329L protein was diluted to a concentration of 2 µg/mL in carbonate buffer solution (pH = 9.6) and used to coat 96-well plates (100 µL/well) overnight at 4°C, and subsequently washed three times with PBST and blocked with PBST containing 5% dry milk for 2 h at 37°C. Thereafter, 100 µL of positive serum obtained from I329L protein-immunized mice and negative serum from unimmunized mice (1:1000 dilution) were used as controls and incubated at 37°C for 60 min. HRP-conjugated goat anti-mouse IgG monoclonal antibody (Beyotime) (1:1000 dilution) was added and incubated at 37°C for 45 min. TMB (Beyotime) was used as the substrate and the reaction was stopped using 2M H_2_SO_4_. Absorbance values were calculated according to the corresponding OD_450_ values.

### 2.8 Determination of the cut-off value

Thirty-five ASFV-positive and sixty -negative pig serum samples were tested using the proposed iELISA method and used to calculate the ROC curve and Youden index for determining the cut-off value of the iEILSA in SPSS software version 19.0 (Lv et al., 2021).

### 2.9 Specificity, sensitivity, and repeatability

Four types of antisera positive for other swine viruses (PRRSV, PRV, CSFV, and PCV2) were detected using iELISA to determine the specificity. The sensitivity of the iELISA was determined by 2-fold serial dilution of ASFV-positive sera in the range of 1:100–1:6400.

Five inactivated ASFV-positive sera and five negative sera were tested using iELISA based on the purified I329L protein from one batch, in which each serum was set as five parallel holes. Purified proteins from the other two batches were used for repeated detection. Following data collection, the coefficient of variation was calculated to verify its stability.

### 2.10 Detection of ASFV antibodies of pig sera

A total of 186 pig serum samples collected from different regions of China analyzed were analyzed using iELISA and a commercial ELISA kit coated with p32, p62, and p72 recombinant proteins (IDvet, Grabels, France). The coincidence rate between the two methods was then calculated.

## 3 Results

### 3.1 Expression, purification, and identification of the I329L protein

**Figure 1:**
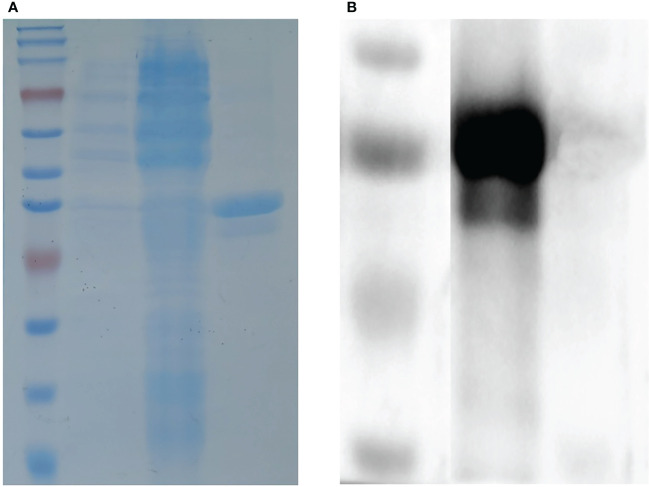
Identification of the recombinant ASFV I329L protein. **(A)** SDS-PAGE analysis of the recombinant ASFV I329L protein. M: protein marker; 1: protein from uninduced transformed (*E*) *coli*; 2: protein from induced transformed (*E*) *coli*; 3: purified protein. **(B)** Identification of the recombinant ASFV I329L protein using an ASFV-positive serum *via* western blot. M: protein marker; 1: purified protein.

The truncated I329L gene was cloned into the pET–28a28a vector and successfully expressed in BL21 cells to yield a protein with a predicted molecular weight of 38 kDa. Further analysis showed the His–I329L protein was present in the inclusion bodies, as shown in **Figure 1A**. The purified recombinant proteins were indicated by the predicted molecular weight in a western blot assay using anti-ASFV pig serum as the positive antibody, as shown in **Figure 1B**.

### 3.2 Antigenicity of the I329L protein

**Figure 2:**
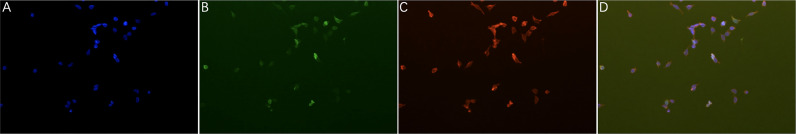
Antigenicity of the recombinant truncated ASFV I329L protein. The full-length I329L recombinant plasmid pCAGGS-EGFP-fI329L was transfected into HEK-293T cells and then incubated with the antiserum (1:500 dilution) of the mouse inoculated with the truncated I329L protein. Red fluorescence showed that the mouse antiserum reacted specifically with the I329L eukaryotic expression protein in 293T cells. **(A)** 293T cells were only stained with DAPI and showed blue fluorescence; **(B)** 293T cells were transfected with the pCAGGS-EGFP-fI329L plasmid and showed green fluorescence; **(C)** The 293T cells transfected with recombinant plasmid were incubated with the positive mouse sera and the goat anti-mouse secondary antibody in order, and observed with red fluorescence; **(D)** The former three images were merged.

The eukaryotic plasmid pCAGGS-EGFP-I329L, encoding the full-length I329L gene, was transfected into HEK-293T cells. Subsequently, indirect immunofluorescence detection was performed to verify the antigenicity of the truncated prokaryotic I329L protein using mouse antiserum (1:500 dilution) against the prokaryotic I329L protein as the primary antibody. The transfected cells showed a large amount of I329L protein-specific red fluorescence (**Figure 2**), indicating that the prokaryotically expressed I329L protein had good antigenicity.

### 3.3 Optimization of the I329L-ELISA

**Figure 3:**
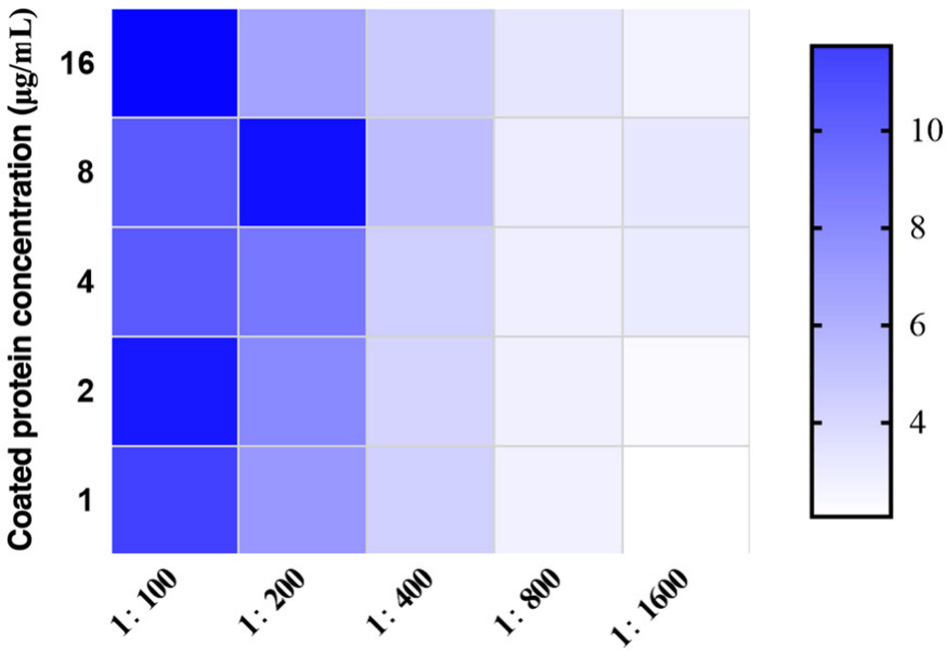
Optimization of the developed ASFV I329L-based iELISA. The optimal working concentration of the coating antigen and pig sera was determined using checkerboard titrations. The ratios of positive to negative reference serum (P/N) are presented in the heatmap. Darker colors indicate higher P/N OD_450_ ratios.

**Figure 4:**
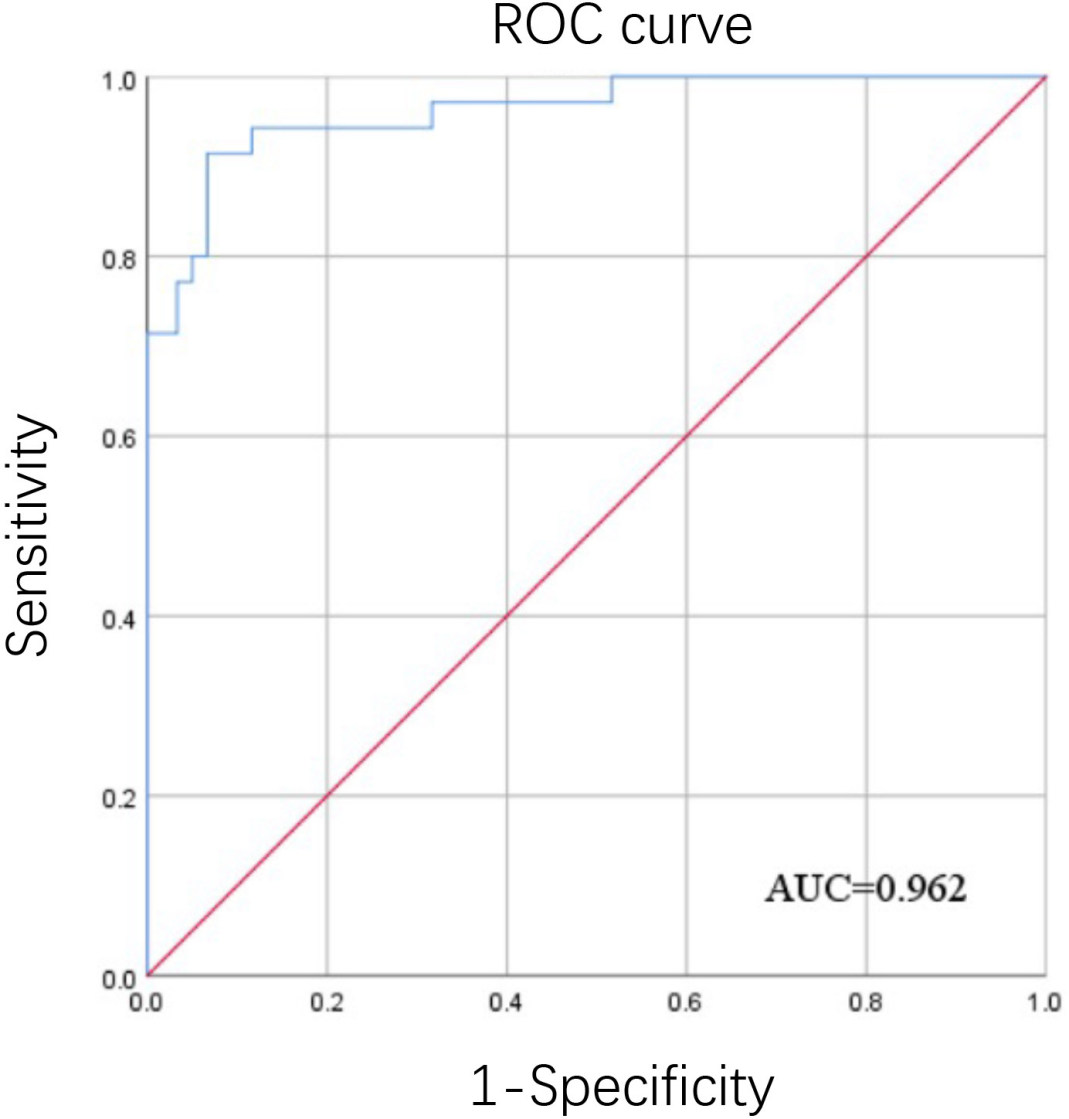
ROC curve of the determination of the iELISA cut-off value. ROC curve analysis was performed using SPSS software using the OD_450_ values from thirty-five positive inactivated sera and sixty negative sera detected by the iELISA.

After detection by chessboard titration method, the optimal concentration of the coating antigen was set as 8.0 μg/mL and the swine sera were diluted 1:200 for the iELISA, as indicated in **Figure 3**. Other optimized parameters were as follows: blocking for 60 min using 5% dry milk, incubation,incubated with the pig serum for 60 min, incubation with secondary antibody for 90 min, and incubation with TMB for 15 min. The cut-off value was analyzed using the ROC curve (**Figure 4**) and Youden index and was determined to be 0.384 at OD_450_ nm. The detection results showed that the iELISA showed good specificity and the antibodies against PRRSV, PRV, CSFV, and PCV2 showed negative in this test. The detection sensitivity of the iELISA was 1:3200. Evaluation of the five selected serum samples revealed inter-assay coefficients of variability (CVs), ranging from 1.90% to 5.21%, and intra-assay CVs, ranging from 4.03% to 7.14%. These data indicated that the I329L-ELISA was repeatable with low variations.

### 3.4 Application of the I329L-ELISA

The I329L-ELISA and IDvet ASF antibody detection kits were used to detect 186 clinical samples, respectively. The results showed that the coincidence rate of the two antibody detection kits was 96.77%, and no significant difference was observed between the two assays (**Table 1**).

**Table 1:** The coincidence tests results between the developed I329L-ELISA and the commercial kit.

Serum samples	I329L-ELISA			Kappa	*P*-value
	Positive samples	Negative samples	Total		
Positive samples tested by the kit	7	1	8	0.684	< 0.001
Negative samples tested by the kit	5	173	178		
Total	12	174	186		
Coincidence rate			96.77%		

## 4 Discussion

ASFV entered China in 2018, and has since become a top priority for the prevention and control of swine diseases in China owing to its high lethality, which has caused substantial economic losses to China’s pig production and related industries. In recent years, China has made considerable progress in the prevention and control of ASF through large-scale culling and strict biosecurity prevention and control measures. However, ASFV has spread nationwide and currently shows multipoint dissemination and still has a high risk of spreading (Zhao et al., 2022). Timely detection of viral infection is essential for culling of affected pigs to stop the spread of the virus. In the context of pig production in China, it is important to establish rapid, efficient, and simple detection methods, especially regular serological screening for ASFV antibodies, so that farming companies can understand the status of ASFV infection in their herds, which helps better prevent and control the disease and minimize the losses caused by an outbreak.

The methods recommended by the World Organization for Animal Health for the detection of ASFV antibodies include ELISA, IFA, and immunoblotting, which are widely used in pig production. Currently, most published references or kits for ASFV monitoring use p30, p32, p54, p62, p72, CD2v, E199L, and other viral proteins as detection targets in domestic and international markets. Among them, the French company IDvet produces an indirect ELISA kit encapsulated with ASFV p32, p62, and p72 recombinant proteins and a competitive ELISA kit coated with ASFV p32 protein. Additionally, an ELISA based on the dual proteins CD2v and p30 was established to differentiate between low-virulence mutant strains with CD2v gene deletion and wild-type viral infections (Yuan et al., 2021). ASFV antibodies were detected using competitive ELISA using specific nanobodies (Nb8) against ASFV p54 based on the phage display technique (Zhao et al., 2022). The blocking ELISAs are mainly based on monoclonal antibodies against ASFV p30 (Yuan et al., 2021) and p54 proteins, which have good specificity, sensitivity, and reproducibility, whereas the double-antibody sandwich ELISA was established based on the ASFV p72 protein. More indirect ELISA methods have been reported, using ASFV pp62, p72/p32, E199L, p30, and CD2v proteins as coated proteins (Zhong et al., 2022); all of which have their respective advantages, such as high sensitivity, specificity, reproducibility, and suitability for early detection.

TheI329L protein is a type I transmembrane protein with 329 amino acids, which is relatively conserved in sequence and is expressed in late-stage viral infection (Correia et al., 2023). Additionally, the I329L protein is homologous to the TLR family of proteins and its intracellular amino acid sequence shows 35% similarity to the BOX1 and BOX2 sequences of TLR3. The extracellular domain contains abundant leucine repeat sequences (LRR) that are important motifs in protein interactions. Previous studies have shown that I329L can antagonize the innate immune activation mediated by TLR3 and inhibit the NF-κB and IRF3 signaling pathways induced by dsRNA/Poly(I:C) stimulation (de Oliveira et al., 2011). In summary, I329L plays an important role in the immune response, can be stably expressed in the prokaryotic system, and induces a strong immunological response.

In this study, to express the I329L protein more efficiently, a specific gene fragment was selected based on its signal peptide properties, hydrophilicity, and B-cell epitope. The transmembrane region sequence was deleted to eliminate the negative effects of the fragment on protein expression. Codon optimization of the *I329L* gene sequence was performed to maintain aa balance between the frequency of codon usage and homologous tRNA, and the effect of rare codons was abrogated by replacing underutilized codons with those commonly used by the host. The results showed that the recombinant I329L protein was expressed in the form of inclusion bodies, which were stable and not easily degraded during the expression and purification processes. The I329L-ELISA established in this study and a commercial ELISA kit was used to test 186 clinical samples simultaneously, and the negative results corresponding to the two assays were consistent at 96.77%. In the former test, 12 out of 186 clinically collected samples showed positive antibodies, a low positive rate. We analyzed the possible reasons for this as firstly, all of the serum samples were collected from pigs showing no clinical signs similar to ASF, so their positive rate was low. Alternatively, these seropositive pigs could be in the state of chronic infection with ASFV and did not yet exhibit clinical signs, for example, a natural attenuated strain has been reported in China (Zhang et al., 2023). Six serum samples with inconsistent results were re-tested using IFA, four of which were IFA-positive and two were IFA-negative, with corresponding positive predicted value of 80% and negative predicted value of 100%. The indirect ELISA established in this study had good specificity and excellent repeatability and reproducibility, with comparable sensitivity to the indirect ELISA method described in previous studies. Therefore, it is well suited for the antibody detection against ASFV and epidemiological surveillance.

## Data availability statement

The original contributions presented in the study are included in the article/**Supplementary Material**. Further inquiries can be directed to the corresponding author.

## Author contributions

Conceptualization, JP; ZS, WQ, HL, CS, and XC performed the experiments. WQ, GW, and JP wrote the draft of the manuscript. All authors contributed to the article and approved the submitted version.

## Supplementary material

Supplementary Figure 1 Amino acid sequence analysis of I329L protein. The amino acid sequences were analyzed for hydrophilicity, hydrophobicity, secondary structure and antigenicity to find regions of high hydrophilicity and antigenicity.
