# Supplementary figures and images for: I329L protein-based indirect ELISA for detecting antibodies specific to African swine fever virus
Source: Front Cell Infect Microbiol. 2023 Jun 7;13:1150042. doi: 10.3389/fcimb.2023.1150042 (PMC10282770; doi:10.3389/fcimb.2023.1150042)

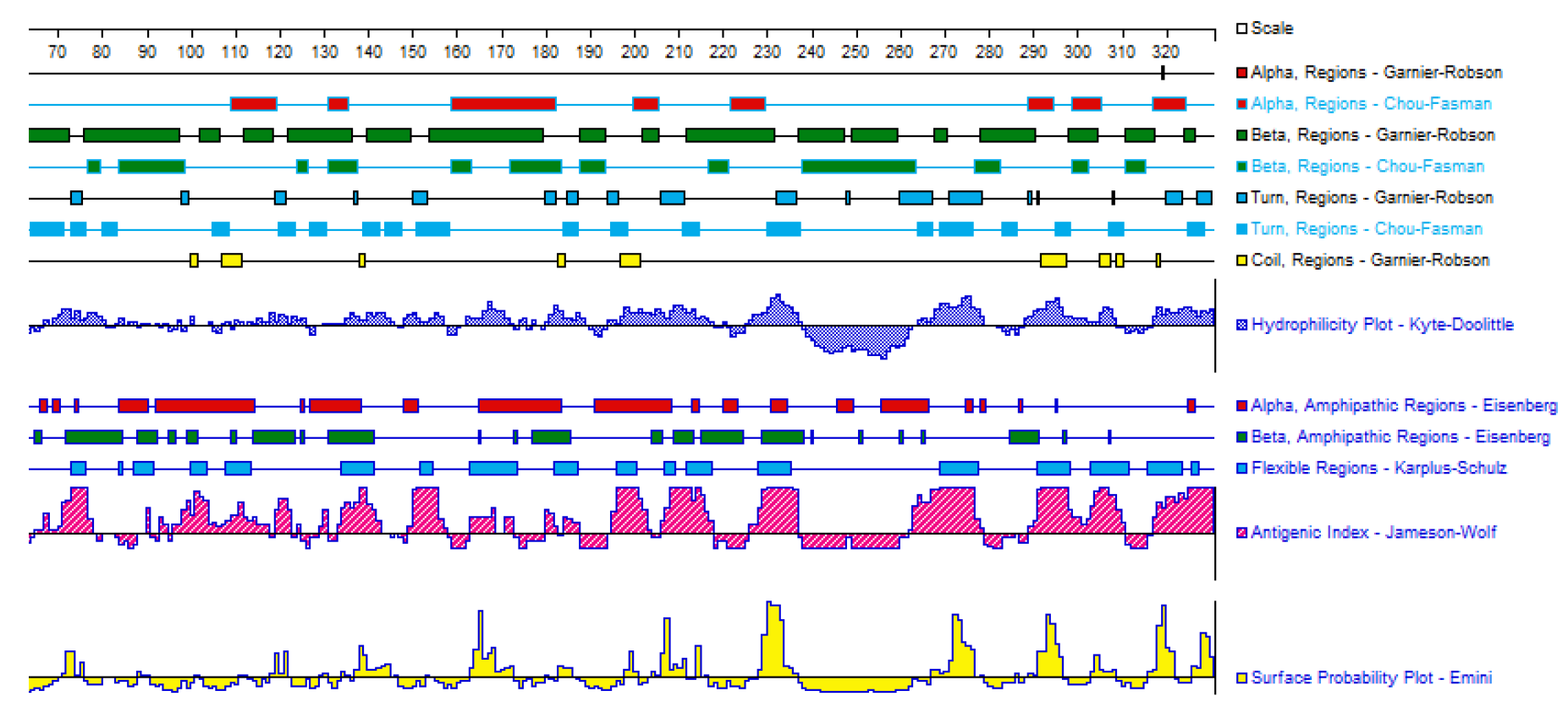

Supplement: Supplementary Figure 1 — Amino acid sequence analysis of I329L protein. The amino acid sequences were analyzed for hydrophilicity, hydrophobicity, secondary structure and antigenicity to find regions of high hydrophilicity and antigenicity. [file Image_1.tif]
